# Functionalized surface of PLGA nanoparticles in thermosensitive gel to enhance the efficacy of antibiotics against antibiotic resistant infections in endodontics: A randomized clinical trial

**DOI:** 10.1016/j.ijpx.2023.100219

**Published:** 2023-11-20

**Authors:** Mona G. Arafa, Hadeel A. Mousa, Mohamed Medhat Kataia, Shehabeldin M., Nagia N. Afifi

**Affiliations:** a Department of Pharmaceutics and Pharmaceutical Technology, Faculty of Pharmacy, The British University in Egypt, Egypt; b Chemotherapeutic Unit, Mansoura University Hospitals, Mansoura, Egypt; c Department of Pharmaceutics in Medical Science, Faculty of Dentistry, The British University in Egypt, Egypt; d Endodontics Department, Faculty of Dentistry, The British University in Egypt, Egypt; e Department of Pharmaceutics, Faculty of Pharmacy, Cairo University, Egypt

**Keywords:** Ciprofloxacin, Nanoparticles, PLGA, Chitosan, Endodontics infection, Thermosensitive gel, Antibacterial drug delivery systems

## Abstract

*Enterococcus faecalis* plays the key role in endodontic infections and is responsible for the formation of biofilm on dentin, which causes a resistance against periradicular lesions treatment, consequently the aim of this study is to use nanoparticles entrapping anibacterial agents coated with chitosan that in authors previous study showed a successful *in vitro* biofilm inhibition, additionally incorporated in thermoresponsive gel.to benefit nanoparticles` small size, and the positive charge of their surfaces that binds with the negatively charged surface of bacterial cell causing its destruction, in addition to the sustained release pattern of the drug based nanoparticles in gel. Therefore, Ciprofloxacin hydrochloride (CIP) encapsulated in PLGA nanoparticles coated with chitosan (CIP-CS-PLGA-NPs), in addition to free CIP, were incorporated in Pluronic® 407/188 to form thermosensitive gels (F1) and (F2), respectively. The thermosensitive gels were tested with regards to rheology, gelling temperature and the release pattern of the drug. A clinical study of the efficacy of F1 and F2 as antibacterial treatments was conducted on patients followed by a comparative studies against CIP and Ca(OH)_2_ pastes in terms of biofilm inhibition assay and total bacterial reduction count and percent.The results revealed that F1 and F2 exhibited gelation temperature of 36.9 ± 0.3 °C and 36.0 ± 0.4 °C, viscosity was 15,000 ± 360.6 and 7023.3 ± 296.8 cP respectively. The cumulative release of F1 and F2 after 72 h was 50.03% ± 0.7345 and 77.98% ± 3.122 respectively. F1 was the most efficient treatment against recurrent *E.faecalis* infection in endodontics that was evident by the highest total bacterial reduction count and percent and biofilm inhibition percent that were recorded in the group treated with F1followed by the group treated with F2. Nanocarriers succeeded in carrying the drug deeply in the root canal and sustaining its effect to abolish the obstinate *E. faecalis* recurrent infection and its biofilm formation.

## Graphical abstract

**Figure:**
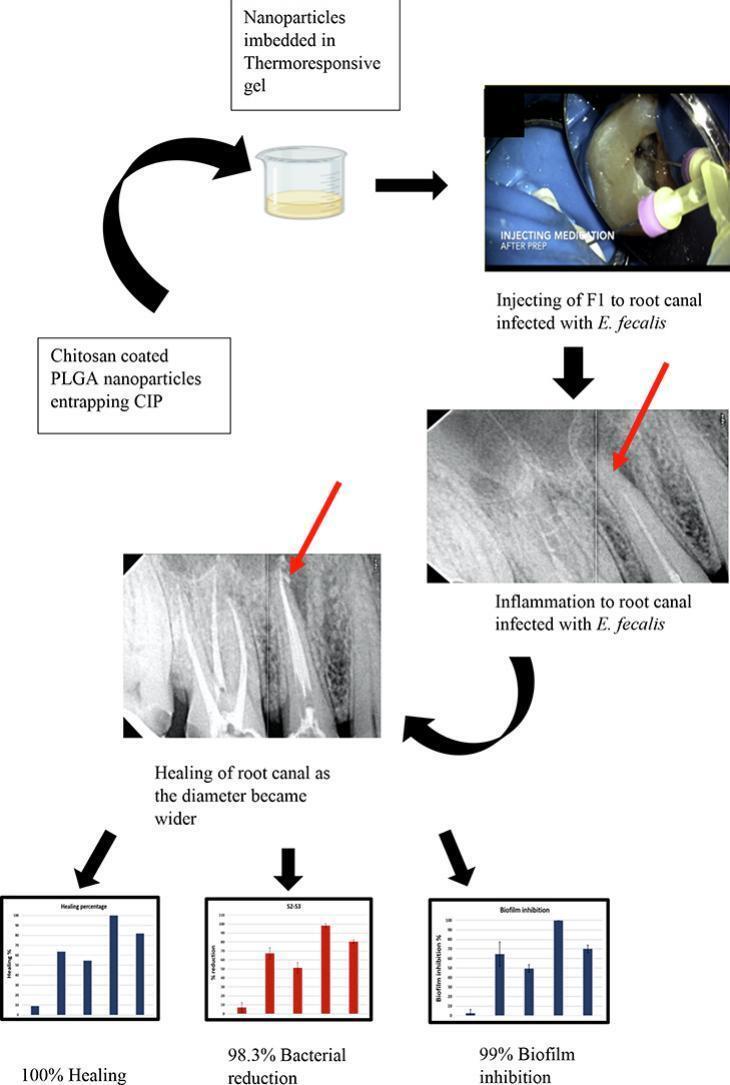

## 1 Introduction

Bacteria and their toxins are reported as the primary etiologic causes of periapical lesions and pulpal necrosis as well. Root canal infections caused by several types of microorganisms such as facultative anaerobic and anaerobic microorganisms, such microbes are mostly found in root canal treatment and cases with post-treatment disease (Wong et al., 2021). *Enterococcus faecalis* is one of the commonly isolated bacteria from failed root canal treatments and resistant infections. *Enterococcus faecalis* is also found in the oral cavity; thus, it can invade root canal and can cause reinfection, also it has the ability to conquer the lymphocytes present in the root canal system, leading to the failure of endodontic's treatment. Furthermore, *Enterococcus faecalis* is capable of forming a biofilm that causes dentin breakdown and can reach dentinal tubules (Ravi Chandra et al., 2015). Bacteria that form biofilm as well as that found in the root canal system can survive without nutrient (Jhajharia et al., 2015; Neelakantan et al., 2017). Bacterial biofilm present in treated root canal is mainly due to the anatomical complexities (Swimberghe et al., 2019). Free bacteria show low antimicrobial resistance compared to that of bacterial biofilm (Sharma et al., 2023; Dutt et al., 2022). While, bacteria that forms biofilm have extracellular polymeric matrix that provides a shielding barrier which plays a key role in the bacterial biofilm resistance (Sharma et al., 2019). Furthermore, dentinal tubules contain bacteria even after instrumentation. In such case, using disinfectant and medicaments directly against pathogens may be become ineffective (Heidar et al., 2020). Although cleaning and shaping reduce bacterial population in the root, complete elimination is hardly achieved because of the complex anatomical system of the root canal (Al-Nazhan et al., 2014; Iandolo et al., 2023). Thus, the use of intracanal medicament between appointments may lead to bacterial elimination before canal filling, in addition of controlling the emerging resistance of the strains side by side with the elimination of smear layer. Intracanal medicament should be non-antigenic, benign and non-carcinogenic, it should be safe and does not cause any side effect on dentin or affects the sealing ability of filling materials, moreover, it should not cause any tooth discoloration. In addition to the above, an ideal medicament should be able to dissolve inactive endotoxins and the pulp tissue (Dutta et al., 2017; Mohammadi and Dummer, 2011).

Several materials were used as intracanal medicaments such as antibiotics, calcium hydroxide, chlorohexidine, steroid, formaldehyde based intracanal medications and recently phytotherapeutic agents such as Propolis, (Wagh et al., 2022; Kumar et al., 2019). Many literature reported that, at the end of the first appointment and after chemo-mechanical preparation was completed, most of root canals still contain feasible microorganisms (Asnaashari and Safavi, 2013; De Castro et al., 2022; Teles et al., 2014; Gomes et al., 2015; Carvalho et al., 2020; Gazzaneo et al., 2021; Pérez et al., 2020; Sathorn et al., 2007; Nandlal et al., 2023), therefore, intracanal medication has contributed a favorable outcome when treating endodontic infections.

Although antibacterial intracanal medication such as ciprofloxacin, have a wide range of spectrum against Gram- negative and Gram- positive bacteria, they have several side effects when taken as conventional oral dosage forms, such as gastrointestinal disorders that include dyspepsia, abdominal pain, vomiting, nausea and diarrhea. There are several side effects that affect CNS as well, such as restlessness, headache, dizziness, drowsiness, insomnia, visual in addition to some sensory troubles, moreover, rare side effects such as hallucinations, psychotic reactions, depression, convulsions, and hypersensitivity reaction. Additional toxic effects of ciprofloxacin include serum creatinine increment, high values of liver's enzymes, hematological disturbances such as eosinophilia, leucopenia, and thrombocytopenia, cardiovascular effects may also occur that include tachycardia, edema, syncope, hot flushes, and sweating were reported (Sharma et al., 2010; Sharma et al., 2017; Grayson et al., 2017; Verma et al., 2013).

In contrast, there are several advantages of local treatment such as; dose increment of medication that leads to high bioavailability of the drug, also avoiding the first pass effect, reducing its side effects and toxicity to other organs (Al-Tahami and Singh, 2007).These advantages have been ignited by using thermoresponsive or *in-situ* gel delivery system which reduces the frequency of dosing, in addition to the ease of administration that improves patient compliance (Al-Tahami and Singh, 2007).

Gels are one of the ultimate local delivery systems of drugs, in which a liquescent phase is controlled with a cross-linked polymeric network, it has large number of hydrophilic groups, as a result; it can hold large amounts of biological fluids and water, besides being biodegradable and biocompatible polymers. Gels are preferred for the treatment of dental infection because they manage to contain a high-water amount that benefits to provide the necessary fluids with a smooth uniformity that is similar to healthy tissue. Although there are several types of gel dosage forms; still thermoresponsive ones are the most convenient and suitable forms to be used as injectable drug delivery systems. Such *in-situ* gels are characterized with increasing the contact time of a drug at the target site, resulting in high bioavailability. Thermoresponsive or *In-situ* gels such as poloxamer 407 and poloxamer 188 exhibit solution to gel phase alteration because of temperature and pH change of their surroundings (Kolašinac et al., 2012; Fàbrega et al., 2009).

In this light, a successful treatment with free CIP and CIP entrapped in PLGA coated with chitosan, and incorporated *in-situ* gels is expected to disrupt the extracellular polymeric matrix of the biofilm by the high antimicrobial resistance bacteria. Furthermore, nanoparticles entrapping antibacterial drugs have been reported lately as an advanced strategy in cleaning root canals (Lode, 2014; Kishen et al., 2008), because of their small size, resulting in great surface area, in addition to their surface charge and polycationic or polyanionic nature which leads to a better interaction with the bacterial cell. Nanoparticles demonstrate remarkable antibacterial and antibiofilm activities (Arafa et al., 2020a), they provide drugs at a sustained rate towards the target site and constrain the drug access to the chosen sites to protect the drug molecule in the systemic circulation. Therefore, a growing need of incorporating CIP in nanoparticles that can penetrate the dentin and dentinal tubules and has antibacterial and antibiofilm effect is highly required. After the success of the *in vitro* antibacterial and antibiofilm action of the nanoparticles in our previous study (Arafa et al., 2018); the same nanoparticles were prepared in a thermo-responsive gel in the current study, to measure their efficacy on patients against recurrent *E. faecalis* infection in endodontics.

The purpose of this work was to prepare CIP loaded in PLGA nanoparticles coated with chitosan and CIP alone as *in-situ* gels, the selected formulae were studied for their antibacterial efficacy on patients having root canal enterococci infection by determining bacterial reduction percent and biofilm inhibition assay, compared with the conventional treatment of CIP and calcium hydroxide pasts.

## 2 Materials and methods

### 2.1 Materials

PLGA’ 50:50 ester terminated molecular weight 38,000–54,000 Da, chitosan 50,000–190,000 Da and PVA molecular weight 31,000–50,000 were supplied by Sigma Aldrich, Berlin, Germany, Ciprofloxacin hydrochloride was gifted from EPICO Company, Cairo, Egypt, Poloxamer (407), molecular weight: 12,600 Da (CAS 9003–11–6) and Poloxamer (188), molecular weight: 8400 Da (CAS 9003–11–6 were purchased from Sigma Aldrich, Germany, Cellophane membrane molecular weight cut out (12000–14,000 Da) was purchased from Spectrum Medical Inc., Raleigh, NC, USA, Dichloromethane was purchased from PioChem, Egypt, Calcium hydroxide in barium sulphate base meta-paste was purchased from Meta Biomed, Korea, Sodium thiosulphate and sodium hypchlorite were purchased from El Gomhorya, Cairo, Egypt, Brain heart agar was purchased from LAB M, United Kingdom, A 96-well microtiter plate was purchased from Costar, Corning Inc., NY, USA, Crystal Violet was purchased from Oxford Lab chem, India, Tryptone soya broth was purchased from OXOID, England, All other solvents were of analytical grade.

### 2.2 Methodology

#### 2.2.1 Preparation of CIP-CS-PLGA-NPs and CIP thermoresponsive gels

Thermo-responsive gelling property is characterized as the main property for poloxamer (407) and (188). Therefore, in order to prepare *in-situ* gels, the cold method was applied. A weighed amount of poloxamer 188 and 407 (30% *w*/w) in a ratio of 21/9 as listed in Table 1 was added to cold distilled water at 4 °C containing our previously prepared formula (Arafa et al., 2018) to form F1, in addition to the same amount of free CIP to form F2, followed by vigorous magnetic stirring, to attain a clear mixtures. The mixtures were kept in the refrigerator overnight where viscous and lucid translucent gels were obtained (Bhandwalkar and Avachat, 2013).

**Table 1:** Composition of poloxamer 407 and 188 thermoresponsive gels.

	Composition of different *in-situ* gels			
Formulae	Poloxamer concentration P407/188 (*w*/w%)	CIP (mg)	PLGA (mg)	Chitosan (mg)
F1	21/9	50	250	50
F2	21/9	50	0	0

### 2.3 Evaluation of the prepared thermoresponsive gels

#### 2.3.1 Measurement of gelation temperatures

Two ml of F1& F2 at 4 °C was poured in a test tube then submerged in a water bath that was maintained at 25 °C Gelation temperature was attained by raising the temperature of the water bath by 1 °C every 5 min, until the gel stops moving upon orienting the test tube. The least temperature is recorded when immobility of the gel was reached is considered to be gelation temperature for the preparation (Swain et al., 2019).

#### 2.3.2 Physical properties of thermoresponsive gels

The formulae were tested for color, homogeneity, texture and pH. The color of the colloidal solutions was observed using white and black sheets in the background (Kurniawansyah et al., 2019).

#### 2.3.3 Rheological study

The rheological property of the prepared *in-situ* gels was determined using Brookfield viscometer (Brookfield Engineering Laboratories, INC, USA), referring to the method mentioned by Liu et al., (Liu et al., 2023). The formulae under study were placed into a small beaker and the spindle of the viscometer was submerged in the gel under investigation, then the spindle was revolved at different rpms (Makwana et al., 2016). The viscosity of F1 and F2 were measured at 10, 20, 50 and 100 rpm at 37 ± 0.5 °C.

#### 2.3.4 *In-vitro* drug release of thermoresponsive gels

*In-vitro* release was investigated using the membrane diffusion technique. A cellophane membrane was gently submerged in phosphate buffer saline (pH 7.4) and then fixed by a piece of latex. After spreading a certain amount of F1 and F2 equivalent to 20 mg of CIP on the cellophane membrane, 1.5 ml of phosphate buffer saline was added to each tube (Bhandwalkar and Avachat, 2013). Then, they were immersed in beakers containing 50 ml phosphate buffer saline at 37 ± 0.5 °C. The water bath (Wise bath, DAIHAN scientific Co. Ltd., Korea) was shaken at 25 strokes per minute. Samples were pulled out and compensated with the same milliliters of phosphate buffer saline at predetermined time points. The released amounts of the drug were analyzed spectrophotometrically at 276 nm using UV spectrophotometer (Jasco v-630, Jasco, Japan), followed by adjusted residual *p*-value for each formula. The p-value was compared with the value of 0.05 alpha level. Statistical analysis was performed using Graphpad Prism.9.

It was reported that poloxamers showed no interference absorbance (Butt et al., 2015). All experiments were done in triplicates.

#### 2.3.5 Kinetic release study

The obtained release data were studied and kinetically analyzed (Siepmann and Peppas, 2011) based on the following models:

a) Zero-order kinetic: C = C_0_ – K_0_t
b) First-order kinetic: log C=log C_0_ – K_1_ t /2.303.
c) Higuchi diffusion kinetics: Q/A = 2 C_0_(D/K_n_)^1/2^t^1/2^.

And for better understanding of the kinetic results and release pattern of the drug Korsmeyer-Peppas model was also applied with “n” value (0.45 < *n* < 0.89).

## 3 Clinical study of CIP-CS-PLGA-NPs and CIP *in situ* gels

**Ethics Approval** The protocol of all clinical work for the current study was executed in agreement with Helsinki Declaration (1996), **in the period between 1st of March 2020 to 15th of March 2020**, after being reviewed and agreed by the Committee of Research Ethics,Faculty of Pharmacy, Cairo University (number of protocol: PI (1643)) and The Ethical Committee of Faculty of Pharmacy, The British University in Egypt (BUE), also the current study was registered at **ClinicalTrials.gov** **registry with Clinical trial ID** **NCT05475444**

### 3.1 Sample size determination

A power analysis was used to calculate the difference between the antibacterial effect of different tested intracanal medicaments under investigation in term of statistics. According to the reported method of Siqueira and Rôças (Rôças et al., 2004), and Schirrmeister et al. (Al-Ahmad et al., 2009); an (α) level type error one of 0.05 (5%), with significant difference *p* ≤ 0.05, and a (β) level type error two of 0.20 (20%), [*i.e.* power of study without error is (80%)], the expected sample size (n) for five groups with different medications was determined as (Iandolo et al., 2023) cases per group. G*Power version 3.1.9.4.was used to calculate the size of sample.

### 3.2 Selection of the patients

CONSORT guidelines for writing randomized clinical trials was used to design the current clinical trial, in addition of being parallel and double blinded trial. The criteria of selecting patients necessitate including 55 patients who have teeth with single root that showed marks and/or manifestation of diseases after treatments with no indication of non-surgical root canal management, following simple randomized procedure by the aid of IBM SPSS V23 (IBM, IBM SPSS Statistics Version 2.0 for Windows USA) statistical analysis software. Participants of each group of the five groups (11 participants per group), were randomly assigned with an allocation ratio of 1:1 through selection of a sealed envelope with a number from (1 to 55).

### 3.3 Inclusion criteria

Single-rooted teeth that were endodontically treated and have manifested post treatment disease exhibiting one or more of the following indications:

- Record of continual acute and/or chronic periapical blisters.
- After one month of prime treating with the formulae, Palpation pain and/or percussion is noticed.
- Radiographic bone loss that may be due to the increased size of an old-existing one or noticed as a new injury.

All patients were enrolled and treated at the endodontic department at the faculty of Dentistry at the British University in Egypt during the period **between 1st of March 2020 to 15th of March 2020.**

### 3.4 Exclusion criteria

- Mutilated teeth indicated for extraction.
- Immuno-compromised patients.
- Patients with a record of oral antibiotics therapy.

### 3.5 Patient classification and administered medication

According to the intracanal medicament used in the first visit:

• First group: (Group I, −ve control) in which root canals were not treated with any medication
• Second group: (Group II,+ ve control): in which root canals were treated with a paste of intra-canal calcium hydroxide
• Third group: (Group III, in which root canals were treated with a paste of CIP with saline, prepared in weight ratio of 4:1 (Madhukumar et al., 2021).
• Fourth group: (Group IV, in which root canals were treated with (F1) CIP entrapped in PLGA coated with chitosan in *in situ* gel).
• Fifth group: (Group V, in which root canals were treated with (F2) free CIP in *in situ* gel).

Groups (III), (IV) and (V), received preparations containing CIP concentration equivalent to 4 mg. Choice of CIP dose was based on 0.2 μg/ml of ciprofloxacin in nanomatrix gel as a minimum bactericidal concentration against *E. faecalis (*Kaushik et al., *2015**)*. Based on the findings in our previous study (Arafa et al., 2020a), 0.5 mg/ml was the effective dose that caused biofilm inhibition.

MK performed the diagnosis and participants enrollment, HA assigned the participants to interventions, SS performed all clinical procedures while MA did the statistics. Both the participants and the statistician were blinded, while the operator could not be blinded due to the nature of the intervention used.

### 3.6 Clinical and microbiological evaluation

#### 3.6.1 Primary visit

**Fig. 1:**
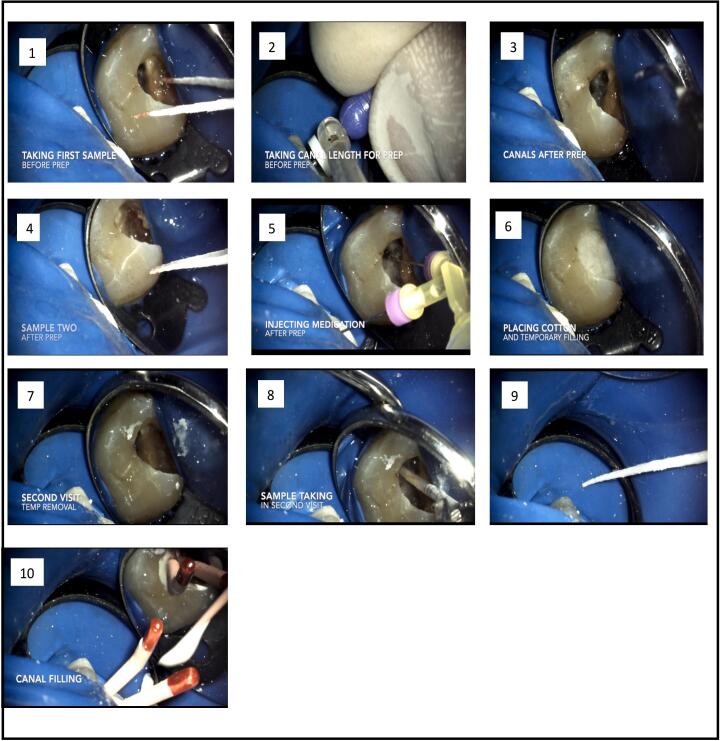
The steps of root canal treatment using medications under investigation.

Treatment protocol was explained to the patients and patients' approval consents were obtained. Fig. 1 is an illustrative diagram that represents the steps of root canal treatment using medications under investigation.

All coronal decay and defective restorations were removed and teeth were restored before rubber dam isolation, the tooth surface was first disinfected, then an access cavity was prepared. Existing root canal filling material was mechanically removed using manual k files #15 and #10 using sterile saline as an irrigant until patency was obtained. Determination of the working length was verified by radiographic and electronic methods. Root canals were soaked with sterile saline, then the walls were scrubbed with small manual files. To obtain the first bacteriological sample (**S1**), a sterile paper point was positioned in the canal farther from the length of the teeth by one millimeter for one minute, the used paper point was equal to the dimension of largest file attaining the determined length of the teeth under investigation, followed by placing the paper point in 1 ml of sterile medium. Fifteen ml of 2.5% sodium hypochlorite irrigant were added to the root canal to complete the preparation process using a gauge 27. A side vented needle of the master apical file (MAF) was decided to be at least of three consecutive sizes after the initial file that binds at the working length, and reveals clean dentin debris. 10 ml of 5% sodium thiosulphate was used to inhibit the action of sodium hypochlorite. Five ml sterile saline was used to flush sodium thiosulphate from the canal, followed by obtaining the second bacteriological sample (**S2**) by placing a sterile paper point distant away from the working length by 1 ml to soak the fluid, for one minute in the canal, which is equal to the dimensions of MAF, then the paper point was sited in 1 ml of the sterile medium.

The patients were chosen randomly according to either of the five groups mentioned before for 7 days. At last, a sterile dressing and coronal restoration was applied under aseptic conditions (European Society of Endodontology, 2006).

#### 3.6.2 Second visit

• An evaluation of the clinical marks and indications was carried out.

#### 3.6.3 Clinical assessment of healing

1. Absence of pain with percussion or palpation.
2. Treated tooth is functional and asymptomatic.

Root canals were obstructed when the patient demonstrated the above two clinical findings. Cleaning, shaping, and treatment were repeated, when the patient showed one or all of the above two clinical findings, followed by scheduling the patient after 7 days.

Before obturation, the intracanal medicaments were washed out of the canals by irrigation with 20 ml of sterile saline. Then the final bacteriological sample (**S3**) was obtained using paper points as described before.

Each sterile paper point of (S1, S2, S3) was sited, in a falcon tube that was previously sterilized and contains one ml of sterile tryptic soy broth supplemented and 0.5% glucose, each tube had a label stated the order of the sample and the type of medication followed by transferring all tubes to the microbiology laboratory in an ice box within 2–3 h from sampling procedure (European Society of Endodontology, 2006).

#### 3.6.4 Microbiological evaluation

##### 3.6.4.1 Total bacterial reduction count and percent

One hundred μl from the collected sample from the groups was added to the brain heart media using sterile inoculum and incubated for 24 h at 37 °C, then the total number of bacterial counts and percent was assessed (Costa et al., 2018; Qamar et al., 2023).

##### 3.6.4.2 Biofilm inhibition assay

A (TSB) complemented with glucose (TSBG) of 0.5% (*w*/w) was used to perform microbial multiplication of the gathered samples (S1, S2, S3) to be assessed quantitatively to detect its ability on developing a biofilm of microbial consortium. Cultures were developed overnight at 37 °C, and diluted in sterile (TSBG) to tone with turbidity accustomed of 0.5 McFarland, which equals 1.5 × 10^8^ CFU/ml. (TSBG) was used to dilute the bacterial suspensions to give 1.5 * 10^8^ CFU/ml cell count. Two hundred μl of the bacterial suspensions were transferred under aseptic conditions to three wells of a sterile 96-well microtiter plate, and incubated for 24 h at 37 °C. then, the cultures were isolated and plates were rinsed triplicate using 200 μl of 0.1% saline was used to remove non-adherent cells. Air drying was carried out to plates positioned in a vertical position, then the biofilms were tainted using 0.1% *w*/*v* crystal violet solution of 100 μl/well for 2 min., followed by decantation of the crystal violet solution, then 200 μl phosphate buffer saline was used twice in rinsing the biofilms. One hundred μl of a mixture of 20% *v*/v acetone and 80% v/v ethanol was used in each well to wash the adherent solution of crystal violet followed by incubating the plates for 20 min at 25 °C. Finally, a microplate reader (Microplate reader (ELX 800), Biotek, VT, USA) (Sabrah et al., 2013; Ong et al., 2017; Zhang et al., 2013) was used to determine the absorbance at562 nm.

### 3.7 Statistical analysis

For comparing the effect of drug on *E. faecalis* bacterial count percent reduction at each evaluation time, One-way ANOVA followed by Tukey's post-hoc test were performed, and it was also used to compare the different treatments effect on biofilm inhibition. Tukey's post-hoc test was used to compare *E. faecalis* percent reduction between different groups. Chi-square was conducted for statistical analysis to compare the frequency of healing between the different five groups. It was followed by adjusted residual *p*-value of chi distribution for each group. The p-value was compared with the value of 0.05 alpha level. IBM SPSS Statistics Version 2.0. was employed to perform the statistical analysis, additionally, mean and standard deviation (SD) were used to present the obtained data.

## 4 Results & discussion

### 4.1 Preparation of *in situ* gels

The prepared formulae composed of 30% *w*/w 407/188 poloxamer mixture in 21/9 ratio, affected the physical characteristics and attribute of the *in-situ* gels as shown from their evaluation.

### 4.2 Evaluation of the prepared *in-situ* gels

#### 4.2.1 Measurement of the gelation temperatures

Gelation temperature is defined as the temperature at which liquid colloids change to gel. A gelation temperature range which is appropriate for such change is between 33 and 37 °C. The prepared formulae of P407/188 in 21/9 (*w*/w%) of F1 and F2 were investigated for their gelation temperatures. F1, F2 exhibited gelation temperature of 36.9 ± 0.3 °C, and 36.0 ± 0.4 °C respectively as shown in Table 2. This indicated that the formula containing nanoparticles had higher gelation temperature than the formula that contains drug alone. It was reported that P407 solution succeeded to form gel below 25 °C, while 40% P188 solution succeeded to form gel at 50 °C (Ban et al., 2017).

**Table 2:** Gelation temperatures of *in situ* gels.

Formula	Poloxamer concentration P407/188 (*w*/w%)	Gelation temperature ^°^C
F1	21/9	36.9 ± 0.3
F2	21/9	36.03 ± 0.4

The results obtained were in agreement with Banet al., (Ban et al., 2017) who stated that by increasing the concentration of P407 and decreasing that of P188, the gelation temperature was around 37 °C. Also, Garala et al., (Garala et al., 2013) stated that gelation temperature of a liquid gel composed of a mixture of poloxamer 407 and P188 is a concentration dependent. Spherical micelles with a core composed of dehydrated PPO core and a shell composed of hydrated swollen PEO chains (Bodratti and Alexandridis, 2018) were formed based on attaining a certain value of temperature and concentration of the used polymer, that enabled the molecules of the poloxamer in aqueous solution to self-assembly. The temperature dependent gelation of poloxamer 407 and 188 could also be explained by configuration changes, as the exhibited zigzag configuration may be converted to a closed packed molecules, forming a more thick gel as temperature of poloxamer was increased (Park and Song, 2011).

#### 4.2.2 Physical properties of *in situ* gels

Physical properties of the selected formulae were tested for color, homogeneity, texture and pH. F1 color was yellow due to the presence of chitosan, while F2 was white, both were smooth and homogenous. The pH of F1and F2 were 6.81, 6.54 respectively.

#### 4.2.3 Rheological study

Rheology describes distortion of solid and the flow pattern of liquid. Rheology determines viscosity, which designates the resistance of a fluid to flow. In the light of viscosity; Fluids are divided into Newtonian and non- Newtonian systems. It was found that Newtonian systems are liquids that have constant viscosities independent of shear (Reddy and Ahmed, 2014), while, non- Newtonian systems are liquids that exhibit a change in viscosity with shear. Not all non-Newtonian Fluids behave in the same way when stresses are applied. The behavior of non-Newtonian fluids has important implications upon stress, as viscosity of some liquids may increase while that of others may decrease. This could be explained as follows: An increment in viscosity when stress is applied is due to the increment of shear thickening of certain liquids, on the contrary of some other liquids when stress increases, their viscosity decrease due to the decrease of their shear thinning properties. Dilatant fluids exhibit shear thickening character, where viscosity increases by increasing the rate of applied shear stress, meanwhile, pseudoplastic fluids show shear thinning behavior where decrease in viscosity is accompanied with increasing the rate of applied shear stress (Sochi, 2010).

**Fig. 2:**
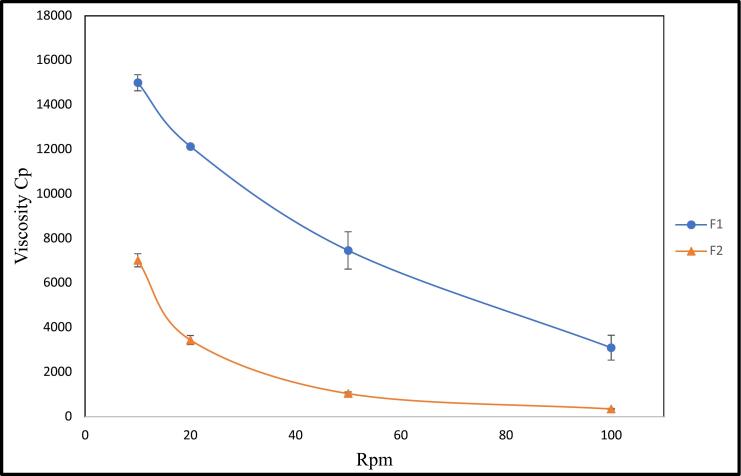
Viscosities of F1 and F2 at different shear rates.

The results in Table 3 as well as, Fig. 2 showed that, F1 recorded a higher viscosity than that of F2, this is probably due to the presence of nanoparticles embedded in *in situ* gel that may bind with the cross linked reticular poloxamer gel resulted in increased viscosity. Also, all thermoresponsive gel formulae showed a characteristic shear thinning flow; they became less viscous as the shear rate increases, which facilitated the flow of the formulations. This can be justifies based on the theory states that, by the least increment in the shear rate, along the direction of their flow; the polymer chains arrange themselves along their axes in parallel to each other, consequently a reduction of any internal resistance was achieved, accordingly the viscosity was decreased (Fathalla et al., 2017). The viscosity of F1 at 10 rpm was (15,000 ± 360.6 Cp), however, further increase in the shear rate till it reached 100 rpm led to a significant decrease in viscosity with (*P* < 0.05) till it was reached (3100 ± 556.8 Cp). Also, F2 showed viscosity of 7023.3 ± 296.8 Cp at 10 rpm that significantly decreased to 346.7 ± 23.6 Cp at 100 rpm. F1 and F2 attain more fluidity, which proved their flow properties. These results were in accord with Ullah et al., (Ullah et al., 2021) who stated that, gels containing Poloxamer 407 were pseudoplastic; therefore, when shearing stress increased; their viscosity decreased (Ullah et al., 2021). These results were in consonance with El- Pereira et al., (Pereira et al., 2013) who studied the properties of poloxamer thermos-responsive gel and indicated that they were pseudoplastic. (Pereira et al., 2013).

**Table 3:** Viscosities of *in situ* gels at different shear rates (rpm).

	Viscosities at different shear rate (Cp)			
Formula	10 rpm	20 rpm	50 rpm	100 rpm
F1	15,000 ± 360.6	12,050 ± 256.6	7466.6 ± 838.6	3100 ± 556.8
F2	7023.3 ± 296.8	3446.7 ± 201.1	1037.33 ± 74.8	346.7 ± 23.6

#### 4.2.4 *In-vitro* drug release of *in situ* gels

**Fig. 3:**
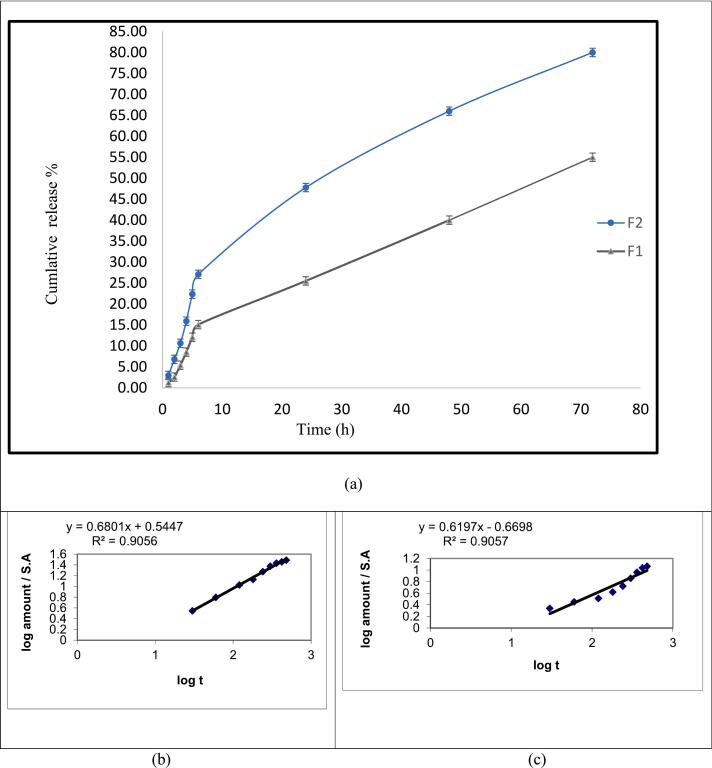
(a) Drug release of CIP from F1 and F2, and Korsmeyer-Peppas kinetics of (b) F1and (c) F2.

The results in Fig. 3 showed that, the cumulative percent of drug release from F1 and F2 in the first 24 h were 25.51% ± 0.5265 and 47.77% ± 1.324, while after 72 h were 50.03% ± 0.7345 and 77.98% ± 3.122 respectively, with significant difference (*P* < 0.0001), alpha = 0.05. This indicated that the *in-situ* gels hindered the drug dissolution and consequently delayed CIP release. In addition to the effect of the gelation temperature that increased the viscosity of the gel, due to the presence of hydrogen bonds of Poloxamer 407 molecules, which form closely fit gel. Moreover, the delay in the drug release pattern may be due to the interaction between CIP and poloxamer 188 (Fathalla et al., 2017). Subsequently, the delay in both gel solubilization followed by drug diffusion delayed the rate of the release of CIP (Sochi, 2010; Mansour et al., 2008). This was in good agreement with Vangala, et al., (Fathalla et al., 2017) who stated that the high P407 concentration and low P188 concentration; the drug release of was only 50% after 12 h. Furthermore, the release of CIP from CS-PLGA-NPs was almost 22% lower than that of CIP alone in *in-situ* gel within a period of 72 h, this may be due to the chitosan coating and PLGA that yielded a thick gel with increased viscosity which reduced CIP release, since the dissolution time was prolonged and drug diffusion through the gel lattice persisted (Fathalla et al., 2017).

#### 4.2.5 Kinetic study

The kinetics analysis of *in vitro* release of F1 and F2 was carried out using linear regression method. The preference of mechanism was based on the correlation coefficient (R^2^), where the preferred selection criteria of the release mechanism is the highest recorded correlation coefficient. The results in Fig. 3 and Table 4 revealed that the pattern of F1 and F2 release were best explained by Higuchi kinetics, the linearity of F1 was R^2^ = 0.985 while of F2 was R^2^ = 0.991, and non Fickian diffusion, this means that the release pattern was dependent on the diffusion erosion of the CIP drug from the polymers of PLGA coated chitosan and *in-situ* gel as well (Arafa et al., 2020b; Pandit et al., 2017). Also, it was suggested that the drug was released through the extra micellar aqueous channels, moreover, to the superficial surface of poloxamer cross-linking system of the gel matrix. The R^2^ values are found in Table 4. The results obtained were similar to those obtained by Arafa et.al (Arafa et al., 2018; Arafa and Ayoub, 2017).

**Table 4:** Kinetics release data of *in-situ* gels.

Formula	Model			
	Zero Order (R^2^)	First order (R^2^)	Higuchi diffusion model (R^2^)	Korsmeyer-Peppas “n”
F1	0.911	0.9103	0.9462	0.68 non Fickian
F2	0.876	0.944	0.9625	0.61 non Fickian

## 5 Clinical and microbiological evaluation

Assessment of the clinical findings was done at the beginning of the second visit.

The statistical analysis was performed on (healed participants and healing percent), bacterial reduction count percent, and biofilm inhibition percent. All participants received the intended interventions and were analyzed for all the outcomes.

### 5.1 Healing process

**Fig. 4:**
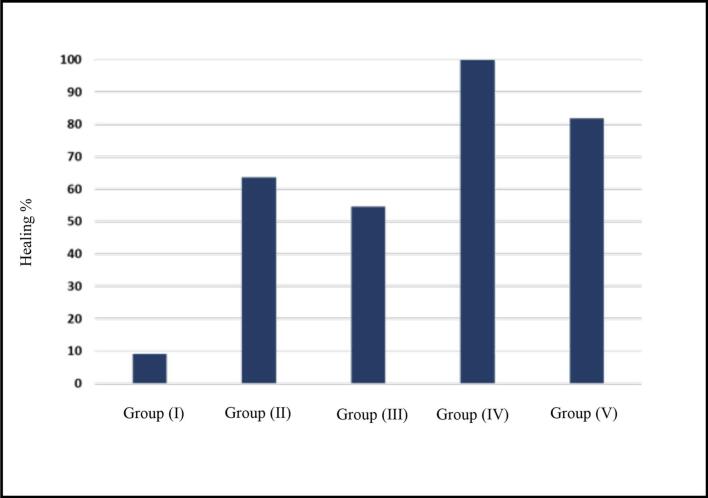
Bar chart of the healing percent in all groups of patients.

**Fig. 5:**
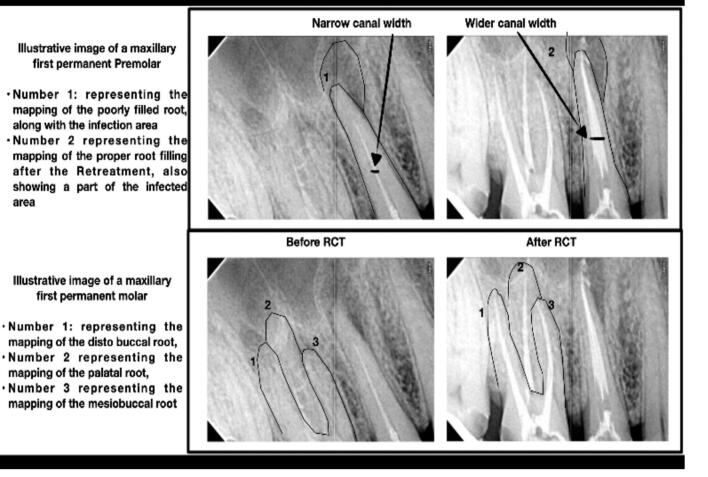
X-ray image of root canal of a patient before and after treatment with F1.

As shown in Table 5, Table 6 and Fig. 4, the best clinical outcome was evident in patients treated in Group (IV), followed by Group (V), Group (II), then Group (III), while the least healing percent was recorded in Group (I) as no treatment was used. The results revealed that there was a significant difference in the healing percent between the five groups. Group (IV) that received chitosan coated PLGA nanoparticles encapsulating ciprofloxacin in *in-situ* gel showed the highest healing percent, as was confirmed in Fig. 5 that showed the X-ray investigation of the root canal of a patient before and after treatment, where the diameter of root canal before treatment was narrow due to infection, while after treatment, it was clear that the diameter of root canal became wider due to healing and biofilm reduction.

**Table 5:** Demographic data of the participants.

		Group (I)	Group (II)	Group (III)	Group (IV)	Group (V)	P-value
Gender	Male n/%	5/45%	7/63%	6/55%	5/45%	6/55%	1.00
	Female n/%	6/55%	4/37%	5/45%	6/55%	5/45%	
Age	Mean ± SD	24 ± 5.3	27 ± 4.1	30 ± 2.9	23 ± 4.6	30 ± 5.2	1.00

**Table 6:** Statistical analysis of the healing percent.

Healing		Group (I)	Group (II)	Group (III)	Group (IV)	Group (V)	P-value
Number of participants	Healed	1	7	6	11	9	<0.0001⁎
	Not healed	10	4	5	0	2	
Healing percentage		9% ^c^	63.63%^b^	54.54%^b^	100%^a^	81.81%^b^	<0.0001⁎

Means with different superscript lowercase letters within the same row are significantly different at P ≤ 0.005.

⁎ : Significant at P ≤ 0.005.

This might be due to the sustained release of CIP, which ensured the presence of CIP during the whole phases of the healing process, in addition to the long residence time of the formula due to the viscosity of the thermoresponsive gel, moreover, the presence of chitosan which had antibacterial and antibiofilm effect that initiated the healing process.

### 5.2 Total bacterial reduction count and percent

**Fig. 6:**
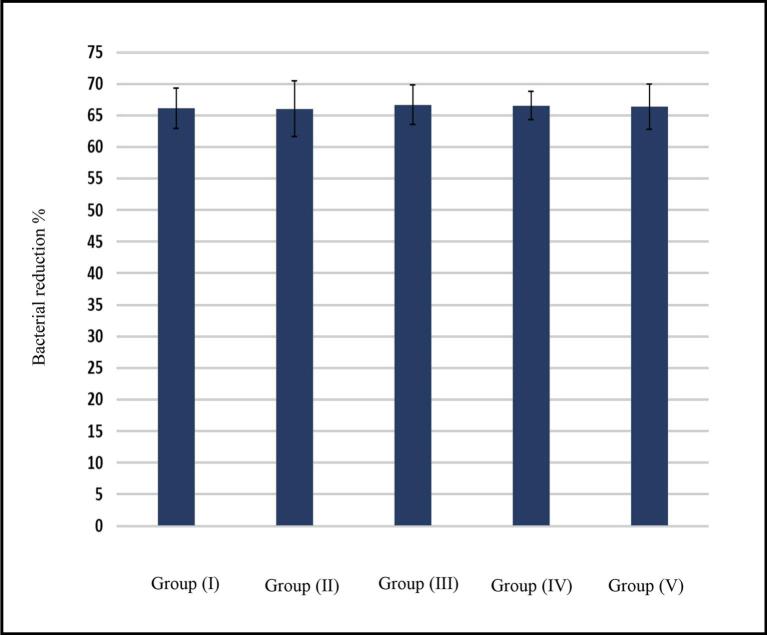
Total bacterial reduction percent after cleaning and shaping (S1-S2).

**Fig. 7:**
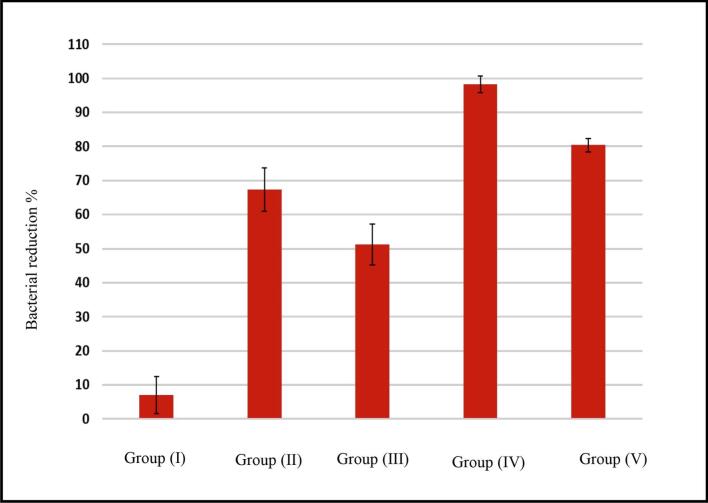
Total bacterial reduction percent after treatment (S2-S3).

High bacterial counting was found in all S1 samples ranged from 4.7 to 7.4 × 10^6^ CFU/mL. After cleaning and shaping S2 ranged from 2.3 to 3.2 × 10^5^ CFU/mL, whilst after adding the medication S3 ranged from 0 to 1.2 × 10^3^ CFU/mL (Arias-Moliz et al., 2012). The bacterial reduction percent after cleaning, shaping (S1-S2) and after medication (S2-S3) are shown in Table 7 and Fig. 6, Fig. 7.

**Table 7:** The reduction in bacterial count percent in terms of shaping and cleaning (S1-S2) and after treating with F1 and F2 (S2-S3).

	Group (I)	Group (II)	Group (III)	Group (IV)	Group (V)	P-value
S1-S2	66.12 ± 3.16^a^	66.04 ± 4.38^a^	66.69 ± 3.14^a^	66.54 ± 2.26^a^	66.32 ± 3.57^a^	0.966
S2-S3	7.0121 ± 5.44^e^	67.4297 ± 6.31^c^	52.089 ± 5.99^d^	98.3976 ± 2.41^a^	80.0514 ± 1.90^b^	<0.001⁎

Means with a,b,c,d and e letters within the same row are significantly different at P ≤ 0.005.

⁎ : Significant at P ≤ 0.005*.*

Regarding the effect of cleaning and shaping alone; there was no significant difference in the bacterial reduction percent among different groups (*p* > 0.005) as shown in Fig. 6. Regarding the effect of intra canal medication alone which was indicated by the difference between (S2-S3), all the five groups were statistically different (*p* < 0.005).

As shown in Fig. 7 the highest total bacterial reduction percent was recorded in Group (IV), followed by Group (V), then calcium hydroxide Group (II), followed by Group (III), while the lowest reduction was recorded in Group (I). Statistical analysis of the obtained results showed that the differences were statistically significant *p* < 0.005.

The results proved that F1 has the highest antibacterial and antibiofilm effect followed by F2, this was due to the effect of chitosan that had a synergistic antibacterial effect (Arafa et al., 2020a; Kariminia et al., 2016; De Queiroz Antonino et al., 2017). Moreover, nanoparticles enhanced the drug penetration to the dentinal tubules and consequently enhanced the antibacterial effect of the drug. Furthermore, drug had high residence time at the target site because of the viscosity of the gel. In addition to the effect of PLGA, CS and *in-situ* gel that sustained the drug release as mentioned before and was in accordance with the outcomes of earlier studies (Makwana et al., 2016; Lu et al., 2019).

### 5.3 Biofilm inhibition assay

**Fig. 8:**
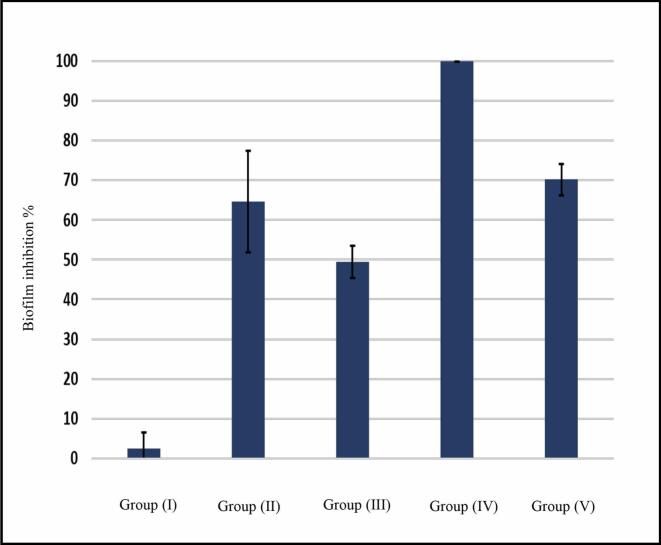
Biofilm inhibition percent.

The biofilm inhibition percent of the five groups were illustrated in Table 8 and Fig. 8, as Group (IV) showed the highest biofilm inhibition followed by Group (V), Group (II), Group (III) and finally Group (I). There was a significant difference between the five groups. F1 showed a 99% biofilm inhibition followed by F2 that showed 70%, then CIP past that recorded 64% inhibition, followed by the least value of 49.49% of Ca (OH)_2_. This was due to the entrapment of CIP in PLGA nanoparticles coated with chitosan incorporated in *in-situ* gel that had antibiofilm effect, which was proven in our previous study (Arafa et al., 2020a). It was proven that PLGA nanoparticles sustained the release of the drug (Kariminia et al., 2016), also *in-situ* gels were able to increase the residence time of the formulae at the root canal, in addition to the effect of chitosan as antibiofilm that gives privilege to sustain the release of the drug as well (Arafa et al., 2020a; Nair et al., 2018; Abosabaa et al., 2021; Bayoumi et al., 2021; Arafa and Ayoub, 2018).

**Table 8:** Statistical analysis of biofilm inhibition percent.

	Group (I)	Group (II)	Group (III)	Group (IV)	Group (V)	P-value
Biofilm inhibition %	2.4183 ± 4.18^d^	64.56 ± 12.82^b^	49.49 ± 4.00^c^	99.96 ± 0.06^a^	70.19 ± 3.96^b^	<0.001⁎

Means with different superscript lowercase letters within the same row are significantly different at P ≤ 0.005.

⁎ : Significant at P ≤ 0.005.

## 6 Conclusions

Poly lactic co-glycolic acid nanoparticles coated with chitosan which highly entrapping Ciprofloxacin hydrochloride, then incorporated in *in situ* gel, were effectively developed and successful in terms of controlled release and endodontic drug targeting delivery as well as achieving higher antibacterial and anti-biofilm activities with significant healing findings than other formulations.

The strength of this study lies in randomized clinical trial confirmed the least systematic error associated with the minimum confounding factors such as age range, therefore the current study is providing precise and genuine information about the current intervention of using antibiotic based nanoparticles in the form of thermoresponsive gel to enhance the efficacy of antibiotics against antibiotic resistant infections in endodontics. Furthermore, it would be of interest that a future investigation will be carried out using the current study outcomes to establish a correlation between short and long-term periapical healing.

## Funding

This work was funded by the authors of the article.

## Author statement

Conceptualization, Visualization, supervision, reviewing, writing and editing were conducted by Mona.G.Aarafa; Investigation and writing original draft were carried out by Hadeel A. Mousa. Conceptualization and supervision was conducted by Nagia N. Afifi, the clinical work of endodontics was conducted by Mohamed Medhat Kataia, Shehabeldin M All authors have read and agreed to the published version of the manuscript.

## List of chemical compounds studied in the article

Ciprofloxacin hydrochloride, EPICO Company, Cairo, Egypt.

Poly (D,L lactide-*co*-glycolide) ‘PLGA’ lactide: glycolide 50:50 ester terminated molecular weight 38,000–54,000 Da, Sigma Aldrich, Berlin, Germany.

chitosan low molecular weight 50,000–190,000, Sigma Aldrich, Berlin, Germany.

Poly vinyl alcohol molecular weight 31,000–50,000 Sigma Aldrich, Berlin, Germany.

Poloxamer (407), molecular weight: 12,600 Da (CAS 9003–11–6) and Poloxamer (188), molecular weight: 8400 Da (CAS 9003–11–6, Sigma Aldrich, Germany.

Calcium hydroxide in barium sulphate base meta-paste, Meta Biomed, Korea.

Sodium thiosulphate and sodium hypchlorite, El Gomhorya, Cairo, Egypt.

Brain heart agar LAB M, United Kingdom.

Crystal Violet Oxford Lab chem, India.

Tryptone soya broth OXOID, England.

## Declaration of Competing Interest

The authors confirm no conflicts of interest in the article.

## Data Availability

No data was used for the research described in the article.
